# Supplementary material for: Causal Relationship between Aging and Anorexia Nervosa: A White-Matter-Microstructure-Mediated Mendelian Randomization Analysis
Source: Biomedicines. 2024 Aug 16;12(8):1874. doi: 10.3390/biomedicines12081874 (PMC11351342; doi:10.3390/biomedicines12081874)
Supplement: Supplementary file 1 [file biomedicines-12-01874-s001.zip › Supplementary Figure/Figure S7/Single SNP plot of MR analysis for ubm-b-1937 to AN.pdf]

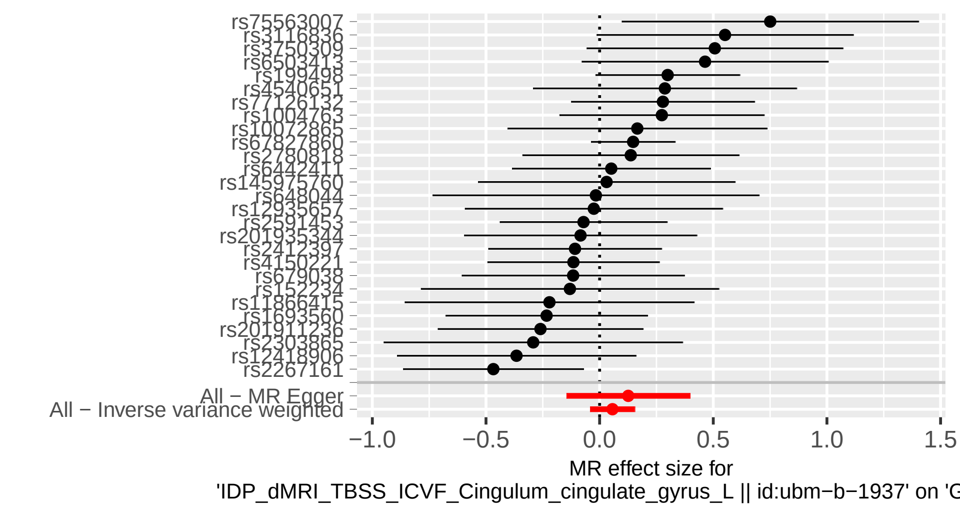
rs75563007
rs3116836
rs3750309
rs6503413
rs199498
rs4540651
rs77126132
rs1004763
rs10072865
rs67827860
rs2780818
rs6442411
rs145975760
rs648044
rs12935657
rs2591453
rs201935344
rs2412397
rs4150221
rs679038
rs152234
rs11866415
rs1693560
rs201911236
rs2303865
rs12418906
rs2267161
All − MR Egger
All − Inverse variance weighted
−1.0
−0.5
0.0
0.5
1.0
1.5
MR effect size for
'IDP_dMRI_TBSS_ICVF_Cingulum_cingulate_gyrus_L || id:ubm−b−1937' on 'G
